# Social and cultural factors perpetuating early marriage in rural Gambia: an exploratory mixed methods study

**DOI:** 10.12688/f1000research.21076.3

**Published:** 2020-01-24

**Authors:** Mat Lowe, Mamsamba Joof, Bomar Mendez Rojas

**Affiliations:** 1 Society for the Study of Women's Health (SSWH), Old Yundum, Kombo North District, The Gambia; 2 Agency for the Development of Women and Children (ADWAC), Kerewan, North Bank Region, The Gambia; 3 Pan American Health Organisation, Tugicigalpa, Honduras

**Keywords:** Early marriage, Gambia, mixed-methods, female adolescents, average age, prevalence, virginity, premarital

## Abstract

**Background:** Over the last two decades, early marriage in the Gambia declined significantly (from 58% to 30%), however this rate is still high. The reasons for the decline but continuing practice of early marriage, despite existing legislation prohibiting child marriage, are not very well understood. Very few studies have been conducted to find out what and how local factors influence decisions about early marriage in the Gambia. More information is therefore needed on underlying reasons for the persistence of early marriage in the Gambia so that program managers can use this information to design strategies to decrease early marriages.

**Methods:** The study was conducted in 24 rural settlements in Lower Baddibu District in the North Bank Region of the Gambia. It was based on a mixed-methods design including a cross-sectional household survey with a sample of 181 female adolescents, focus group discussions with 16 male and female parents, and eight key informant interviews with community-based decision makers.

Focus group discussions and key informant interviews were transcribed verbatim and analyzed using thematic content analysis, while survey data were analyzed using Stata.

**Results:** The study finds that ethnicity and the fear that girls may engage in premarital sex are two important factors associated with early marriage in rural Gambia. In addition, lack of meaningful alternatives to marriage including work opportunities in rural areas may also limit the options and resources available to girls, resulting in early wedlock.

**Conclusions:** These findings suggest that in order to decrease early marriages in rural Gambia, future efforts should focus on understanding and addressing the role of ethnicity in determining marriage patterns and allaying the fear around premarital sex.The findings also suggest a need to provide girls with employment-oriented education including vocational skills which may result into more empowerment and a delay in marriage.

## Introduction

Early marriage or child marriage, defined as marriage before the age of 18, is perceived as a grave violation of human rights. It is a global problem, even though it is prohibited by the Convention on the Rights of the Child and the Convention on the Elimination of All Forms of Discrimination against Women ^1, 2^, two of the most broadly endorsed international human rights agreements. In the last decade, the practice of early marriage declined worldwide from 25% to 21%, a modest reduction of 15% ^3^.

However, the rate of decline has been slowest in West and Central Africa, the region with the highest prevalence of child marriage ^3, 4^. Within the region, estimates vary from 76% in Niger to 18% in Cape Verde ^5^. In West and Central Africa, four in ten girls ^6^ marry before the age of 18, and one in three marries before age 15. At this rate, with the growing population of girls in the region, the number of child brides in West and Central Africa is projected to increase from 6.4 million in 2015 to 7.1 million by 2030 ^4^. Boys in the region also marry early, although girls are disproportionately more affected ^7^. Aside from violating human rights, early marriage adversely affects the health, education and employment.

In West and Central Africa, unlike other African regions, child brides often marry older spouses with multiple partners in a polygamous setting ^8, 9^. This makes it difficult for girls to effectively negotiate safer sex, leaving them vulnerable to sexually transmitted infections, including HIV/AIDS ^8, 10^. The sub-region has the highest rates of early motherhood in the world ^11^. Intense pressure on girls to conceive soon after marriage leads to early pregnancies, short birth spacing, and a higher number of children ^6^. In the region, in 2009, approximately 13.4% of women aged 20 to 24 years gave birth before the age of 16 and nearly 31% by the age of 18 ^9^. The link between too-early childbearing and increased risk of maternal and newborn health problems remains unclear. However, it is suggested that biological, behavioral, social and economic factors combined with inadequate access to and use of health services could exacerbate health problems that directly raise the risk of maternal and newborn health problems ^9, 12^.

The physical immaturity of young girls leads to negative health outcomes that contribute to the high maternal and neonatal mortality in the region. Maternal conditions were the second-leading cause of death among adolescents in sub-Saharan Africa in 2004 ^9^. Early childbearing is also associated with other maternal health problems, including obesity, anemia, malaria, sexually transmitted infection, mental illness and obstetric fistula ^9, 13, 14^. The majority of women with obstetric fistula developed this condition as adolescents and experienced severe physical and social consequences. The negative health effects of early pregnancies also extend to the infants born to adolescent mothers. Infant mortality among babies born to mothers under 20 years of age is 73% higher than that among babies born to mothers over 20; these babies also have a higher risk of being stillborn, having low birth weight, being premature, and dying early ^11^. Either way, women who marry too early bear a disproportionate burden of pregnancy-related death and illness compared to their older peers ^9, 11^.

When girls marry early, they cease formal schooling and education because of domestic and marital demands within the home ^15^. This stops them from acquiring knowledge and life skills that would enable them to become productive members of their households and communities ^16^. This has important negative effects. Girls fail to develop social skills and build supportive social networks with their peers and remain isolated in their homes ^17^. In addition, the lack of formal education also has intergenerational effects. It makes it more difficult for girls to access and use information on health for themselves and their children ^18^, which affects the education attainment, nutritional status, and physical health of their children ^16^. Girls whose mothers married early and had no education or were unable to enter the labor market are more likely to marry early, thus contributing to the cycle of poverty in subsequent generations ^16^.

Young married girls face several barriers to entering the labor market. Their entry is delayed due to early and frequent childbearing, a large family size and their roles as primary caregivers. However, because they are also less educated, they are deprived of the opportunity to gain useful skills and knowledge that will increase their lifelong earning potential ^16, 18^. Therefore, they have limited prospects compared with girls with secondary and post-secondary education for employment in the formal labor market ^18^ and are often confined to housework or work in the informal sector. However, the extent to which child marriage affects labor force participation depends on the country or community ^19^. Household poverty and increased vulnerability to economic shocks often follow a lack of engagement in the labor market, but for communities or societies, these conditions may also significantly reduce economic growth ^18, 20, 21^. Not surprisingly, the economic impact of early marriage holds back the economic development of West and Central African countries ^19^.

The reasons why early marriage is so common in West and Central Africa are wide ranging and can be grouped under religion, tradition and culture, poverty, and gender inequalities ^10, 22– 24^. However, there is evidence that research findings from communities cannot be generalized to other countries or even to other communities within the country where the research was conducted ^25^. Rather, because of this inability to generalize it is crucial to use locally derived evidence to understand how and why communities differ in their approach to child marriage so that more sensitive strategies can be developed to address the problem. The purpose of this study, therefore, was to provide a contextualized picture of underlying factors that are perpetuating early marriage in rural Gambian communities.

## Early marriage in the Gambia

A National Child Protection Strategy Plan, which highlighted early marriage as an issue to address, was launched in 2016. Despite this, early marriages are still common in the Gambia which is listed in the top 41 countries worldwide where the prevalence of child marriage is 30% or more ^26^. Historically, early marriages were widespread with 58% of 40–49-year-old marrying before the age of 18. But this declined over time and now an estimated 30 percent of women aged 20–24 marry before the age of 18. Although this decline rate is significant, it is unlikely to be accurate because many women and adolescents of childbearing age are only recently getting birth certificates ^27^. The reasons for this decline are also uncertain although increasing enrolment of girls in school may be a contributory factor ^28^.

Girls are more affected than boys, with a girl-to-boy ratio for marriage before the age of 18 of 43:1 ^29^. Girls most at risk come from poorer rural households with little or no education ^26, 29^. Urban women aged 25–49 tend to marry about two years later than their rural counterparts. For instance, women in Banjul the capital city marry four years later than women in Kuntaur, a rural settlement 21.0 versus 17.0. For women who have secondary or higher education the median age to marry is 22.2 compared to 17.3 for women who have no education. Similarly, women in the wealthiest quintile marry at a median age of 20.8 years whereas women in the lowest quintiles marry at 17.2 years ^29^. Adolescents are aware of contraceptives (91%) but rarely use them (3.3%), so that childbearing begins early in the Gambia ^29^. Almost one in five (18 percent) of adolescent women age 15–19 are already mothers or pregnant with their first child ^29^.

The reasons for why the practice of early marriage still persists in the Gambia despite the initial decline and the effect of the recent 2016 legal reforms to outlaw early marriage ^30^ have received inadequate attention aside from a single published report ^31^. More information is therefore needed on underlying reasons for the persistence of early marriages in the Gambia. But even more importantly no studies have yet been conducted to design interventions based on locally generated research findings that fully characterize contextual factors influencing decisions on early marriage in rural Gambian communities. The primary purpose of this study is to address these gaps and to provide program managers with detailed information on the type and short-term effects of contextually relevant interventions that can be scaled up to change societal attitudes towards accelerating the decline of early marriages in rural Gambia. The study was conducted as part of a larger research project addressing teen pregnancy and early marriage in the Gambia ^32^.

## Methods

### Study design and setting

This study was based on a mixed-methods design that included a cross-sectional household survey, focus group discussions, and key informant interviews as primary data collection techniques. It was conducted in 24 rural settlements in Lower Baddibu District in the North Bank Region of the Gambia. Lower Baddibu District has a population of 17,961 ^33^. It had the second-lowest recorded median age at marriage (17.3 years) in a national survey ^29^.

### Selection and recruitment of study participants

***Cross-sectional household survey.*** The sampling frame for the cross-sectional household survey was drawn from the Directory of Settlement ^33^, which is the census frame. The sample was selected in two stages. First, all the 24 settlements in Lower Baddibu District were randomly selected and grouped under the four main ethnic groups, Mandinka, Fula, Wolof and Serer, with probability proportional to population size age 10 years and above for each ethnic group of settlement ( Table 1). Further stratification by age was done. Those to be interviewed were divided into three age groups: a) 10–19 years; b) 20–39 years; and c) 40+ years. The proportion of the population in the Gambia that falls into each of these groups is estimated at 35% for 10–19; 42% 20–39 and 23% for those aged 40+ ^33^. These proportions were used to determine the number of those to be interviewed within each age group in each ethnic group of settlements ( Table 2). Female adolescents represent 56% (181 out of 320 adolescents) of the total 10–19 years targeted for the survey ( Table 2). Female adolescents (aged 10–19 years) were the main focus of the survey because the practice of early marriage has a greater impact towards young females.

**Table 1.** Sample size determination by probability proportional to population size.

Major ethnic group	Name of settlements	Population size 10+ years	No of respondents	No. of Households
Mandinka	Kerewan Gunjur Suwareh Kunda Kinteh Kunda Janneh-ya Banni Saaba Daru Salam	8561	311	52
Fula	Tallen Fula Choken Fula Toro Bah Yallal Bah Kerr Banno Mbaburr Kunda Foday Biran Njie Kunda Toro Tayam	1965	277	46
Wolof	Amdalai Tallen Wolof Taiba (Jebel Satou) Njawara Tawakaltu Panneh Bah	1449	264	44
Serer	Samba Musa Choken Missira	78	63	11
	**Total**	**12053**	**915**	**153**

**Table 2.** Sample size determination by ethnic group, settlement, number of respondents and age group (in years).

Major ethnic group	Name of settlements	No of respondents	Age group (years)		
			10–19	20–39	≥40+
Mandinka	Gunjur Kerewan Suwareh Kunda Kinteh Kunda Janneh-ya Banni Saaba Daru Salam	311	109	131	71
Fula	Tallen Fula Choken Fula Toro Bah Yallal Bah Kerr Banno Mbaburr Kunda Foday Biran Njie Kunda Toro Tayam	277	97	116	64
Wolof	Amdalai Tallen Wolof Taiba (Jebel Satou) Njawara Tawakaltu Panneh Bah	264	92	111	61
Serer	Samba Musa Choken Missira	63	22	26	15
	**TOTAL**	**915**	**320**	**384**	**211**

When the research team arrived in the selected settlement, a systematic sampling approach was used to select every second household, followed by convenience sampling to select respondents within each household. Two female respondents aged 10- to 19-year were selected. The research team then continued to collect data in the settlement until they obtained the numbers required. However, if the households did not provide the number of respondents required, then the research team subsequently moved to a random new settlement. Interviews were conducted at noon and sometimes in the late evening to maximize the response rate. No more than two individuals per household in the same age group were interviewed to increase representativeness.

***Focus group discussions.*** The participants were selected purposively, and were mainly parents, who were recruited voluntarily with the help of the research team. They were chosen based on their role as household heads with actual or potential relevance and understanding of decision-making processes around early marriage.

***Key informant interviews.*** The participants for the key informant interviews were mainly community-based decision makers. They included village development committee members, a lady councilor, traditional birth attendants, village heads and a religious leader, who were recruited from the four main ethnic groups (Mandinka, Fula, Wolof, and Serer).

### Data collection

***Quantitative data.*** Two types of questionnaires (a Household Questionnaire and a Female Adolescent Questionnaire, both available as *Extended data* ^34, 35^) were used to conduct face to face interviews with interviewees. The Household Questionnaire was used to identify female adolescents eligible to be interviewed with the Female Adolescent Questionnaire. In addition, the Household Questionnaire also collected information on the condition of the dwelling and household amenities and possessions, such as the source of drinking water, type of sanitation facilities, materials used to construct the dwelling, and ownership of various consumer durables, land, and farm animals. The Female Adolescent Questionnaire collected information on characteristics of female adolescents (age, marital status, ethnicity, literacy, current school attendance, education, employment, exposure to early marriage prevention messages, average age of marriage and characteristics of parents). The questionnaires were adapted from a similar study in Ethiopia ^36^ and from the Demographic Health Survey of the Gambia ^29^. They were pre-tested in three settlements in Sabach Sanjal District, which have similar characteristics to Lower Baddibu District, where the study was conducted. The pre-testing allowed revisions and finalization of the questionnaires before they were put to full-scale administration.

***Qualitative data.*** For the qualitative data collection, two focus group discussions that included 16 participants including both male and female parents were conducted in two selected case study settlements (Njwara and Foday Biran). The focus group discussions typically lasted for 45 minutes and were held in either the village health post or community center to avoid distraction. They were conducted in the vernacular languages (Mandinka, Wolof, Fula and Serer) by the main author and the research team, which comprised of four data collectors and two field supervisors. The main author and the research team have a thorough mastery of the four main languages (Mandinka, Wolof, Fula and Serer) used to conduct the focus group discussions. A focus group discussion guide which was developed based on review of existing literature was used to facilitate discussion during focus group discussions (this is available as *Extended data* ^37^). The focus group discussion guide explored perceptions of and attitudes towards early marriage. It was pre-tested with participants that have similar inclusion criteria as those who participated in the focus group discussions. Key informant interviews (KIIs) were also conducted with eight community-based decision makers to obtain in-depth information about issues surrounding early marriage and marriage patterns in their communities. The FGDs and KIIs were digitally recorded using an IC recorder and transcribed verbatim in English language. The data collection process for both the FGDs and KIIs was based on the principle of saturation ^38^.

### Data management and analysis

***Quantitative data.*** Descriptive analysis of survey respondents’ demographic characteristics was first conducted using the means and standard deviations for continuous variables and the frequencies and percentages for categorical variables. Second, multiple logistic regression analysis was used to determine the predictors of early marriage among female adolescents. We used a progressive analytical strategy starting with the calculations of the unadjusted odds ratios. We first ran model 1 in which ethnic groups were adjusted by messages to prevent early marriage. In model 2 we assessed whether the observed associations of model 1 were explained by the presence of selected household characteristics (a proxy of wealth). In models 3 and 4, we evaluated the extent the associations of previous model are explained by female’s education, adolescent parents’ survival status, adolescent’s parent ability to read and write and whether the female adolescent agree that fathers as heads of household and not mothers should arrange for their daughters’ and sons’ marriage. All the analyses were conducted in Stata version 12.0 produced by StataCorp in College Station, TX.

***Qualitative data.*** Data analysis process for the qualitative data involved listening to all sound recording files from focus group discussions and key informant interviews by the main author and the research team before transcription began. After transcription, the transcribed text was read at least three times to make sense of totality. This process of transcription and reading of the transcribed texts allowed identification of thematic categories, which were later developed into major themes. Coding and analysis of all data were subjected to content analysis ^39^, and NVivo 11 Pro was used to manage the data.

### Ethical considerations

Before the start of the study, the main author submitted the study proposal and tools to the Scientific Coordinating Committee (SCC) Medical Research Council (MRC) The Gambia at the London School of Hygiene and Tropical Medicine (LSHTM). The main author was then invited to present at the meeting of the SCC. The SCC provided some inputs on the study proposal and tools following presentation by the main author at its meeting. The revised study proposal and tools in relation to the issues raised by the SCC was re-submitted by the main author and later approved by the SCC. In the study proposal and tools was later submitted and approved by the SCC (SCC 1651v1.1). The SCC then subsequently forwarded the approved study proposal and tools to the Joint Gambia Government/MRC Ethics Committee for further consideration, which later provided ethical approval (SCC 1651v1.2) following minor changes on the informed consent form.

During data collection, informed consent, verbal and written depending on the level of literacy, were obtained from the study participants. Parental informed consent was sought for minors under the age of 18 years. Since the research team was composed of men, permission was also taken from husbands of married female adolescents because culturally, husbands are expected to give permission for other men to speak with their wives ^40^.

Care was also taken to make sure that all questions were asked in a culturally respectful and non-judgmental way. This was achieved through the careful selection and training of data collectors and field supervisors, as well as by the design of the study tools. In all the study communities, courtesy calls were also made to village heads (locally known as “Alkaloes”) to inform them about the purpose of the study and to seek clearance from them. Although this method of obtaining approval is considered customary, it is highly ethical and recommended, since village heads yield much power over their people and territories.

## Results

The results are presented in two sections. First, data on demographic characteristics of female adolescents are presented, followed by factors associated with early marriage. Individual-level responses to questionnaires ^41^, and de-identified summary transcripts of focus group discussions and summary transcript of key informant interviews ^42^ are available as Underlying data. Focus group transcripts ^43^ are available as *Underlying data*.

### Demographic characteristics of female respondents

**Figure 1.**
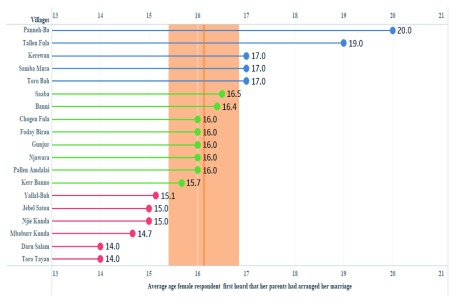
Average age at which a female adolescent first heard that her parents had arranged her marriage. Five villages are not displayed because they have no data on average age at which female adolescent first heard that her parents had arranged her marriage.

Table 3 presents the demographic characteristics of female adolescents in the cross-sectional household survey. Nearly 70% of female adolescents belonged to the Mandinka and Fula ethnic groups and are older than 13 years. About 12% of them are currently or ever married. On average, female adolescents have 4.2 years of schooling and about 45% are currently attending school. The average age at which female adolescents first heard that their parents had arranged for their marriage was 16 years ( Figure 1), which is below the national legal age of 18 years of marriage.

**Table 3.** Demographic characteristics of female adolescents aged 10–19 years.

Variable	Female adolescents n = 181	
	Mean (SD)	%
**Ethnic group**		
Fula		32.5
Mandinka		37.6
Serer		5.1
Wolof		24.9
**Age group**		
10 to 12 years		17.8
13 to 15 years		35.0
16 to 19 years		47.2
**Marital status**		
Currently/ever married		11.7
Never married		88.3
**Years of schooling**	4.2 (4.1)	
**Currently attending school**		
Yes		45.2
**Years of education**		
0 years		41.6
1 to 6 years		21.3
7 to 9 years		27.4
10 to 12 years		9.6
**Activity has spent most of the** **time spent in the last 12 months**		
Going to school/studying		45.2
Housework/child care		40.6
Looking for work		14.2

The vertical orange line is the average age at which the female respondent first heard that her parents arranged her marriage. The orange area represents the 95% confidence interval of the average. The average was 16 years, the minimum value of the average was 15.1 years, and the maximum value was 16.6 years. The horizontal red lines and circles represent villages in which the average age is below the minimum value, the blue lines represent the villages in which the average age is above the maximum value, and the green lines represent the villages in which the average age falls within the average range for this area.

### Factors associated with early marriage

***Factors identified using multiple logistic regression analysis***. Table 4 shows regression analysis results for factors associated with marrying before 18 years among female adolescents. In the unadjusted associations, females in both the Mandinka and Wolof ethnic groups have a higher probability of marrying before 18 years than do females in the Fula ethnic group. Compared with the reference group, females in the Mandinka ethnic group have a 3-fold higher probability of marrying before 18 years, and those in the Wolof ethnic group have a 2.9-fold higher probability; both results are statistically significant at the 0.05 level.

**Table 4.** Regression analysis results for factors associated with marrying before 18 years.

Variables	Unadjusted	Model 1	Model 2	Model 3	Model 4
	OR (95 % CI) ^1^	OR (95 % CI)	OR (95 % CI)	OR (95 % CI)	OR (95 % CI)
Ethnic group (reference: Fula) ^2^					
Mandinka	3.1 ** (1.34 – 7.28)	2.32* (0.85 – 6.32)	2.1 (0.82 – 5.18)	1.8 (0.69 – 4.69)	1.79 (0.66 – 4.84)
Wolof	2.9 ** (1.12 – 7.61)	2.84* (0.85 – 9.47)	3.16 ** (1.17 – 8.48)	3.0 ** (1.07 – 8.61)	2.85 ** (1.0 – 8.15)
Number of messages of preventing early marriage seen or heard by female youth (Reference: Not heard or seen messages)					
From one source	0.72 (0.28 – 1.82)	0.58 (0.18 – 1.83)	0.58 (0.18 – 1.86)	0.71 (0.21 – 2.39)	0.65 (0.18 – 2.27)
From two sources	1.23 (0.42 – 3.58)	0.67 (0.19 – 2.32)	0.65 (0.18 – 2.29)	0.83 (0.22 – 3.10)	0.93 (0.23 – 3.64)
From three to five sources	0.70 (0.18 – 2.61)	1.76 (0.18 – 16.6)	2.23 (0.22 – 21.9)	3.35 (0.32 – 35.4)	2.94 (0.24 – 35.1)
Selected household characteristics (reference: Households have none of the characteristics)					
Households with Electricity AND Piped water into dwelling AND Ventilated improved pit latrine	2.20 (0.78 – 6.17)		2.91 ** (1.13 – 7.48)	2.54 (0.96 – 6.73)	3.24 ** (1.13 – 9.31)
Electricity AND Piped water into dwelling AND Ventilated improved pit latrine AND Walls made of bricks/cement/ cement blocks AND Roof made of corrugated iron AND Floor made of cement/bricks/ceramic	4.05 ** (1.07 – 15.3)		5.15 ** (1.47 – 18.0)	5.39 ** (1.49 – 19.5)	6.97 ** (1.79 – 27.1)
Years of adolescent female’s schooling (reference: 8 to 12 years)					
0	2.20 (0.66 – 7.35)			0.86 (0.33 – 2.20)	0.82 (0.31- 2.19)
1 to 4	13.5 ** (1.45 – 18.6)			4.31 (0.47 – 39.2)	4.36 (0.47 – 40.2)
5 to 7	2.19 (0.60 – 8.02)			2.19 (0.69 – 6.92)	1.76 (0.54 – 5.78)
Mother is alive (yes)	0.74 (0.09 – 6.14)				2.11 (0.39 – 11.4)
Father is alive (yes)	1.84 (0.66 – 5.1)				1.36 (0.47 – 3.93)
Father can read and write (yes)	2.60 (0.58 – 11.6)				0.78 (0.25 – 2.44)
Mother can read and write (yes)	3.05 (0.39 – 23.9)				2.18 (0.43 – 11.1)
The female adolescent agree that fathers as heads of household and not mothers should arrange for their daughters’ and sons’ marriage	2.47 (0.80 – 7.60)				1.64 (0.64 – 4.25)

**The results are statistically significant at 0.05 level. 1= Unadjusted odds ratio; 2= The Serer ethnic group has no enough observations to be included in this logistic regression analysis

In Model 1, we entered the ethnic groups simultaneously and whether the respondent was exposed to messages regarding the prevention of early marriage, as classified by the number of sources (e.g., radio, TV). After the number of messages received was held constant, females in the Mandinka and Wolof ethnic groups had a 2-fold greater probability of marrying early than did females in the Fula ethnic group.

In Model 2, we evaluated the association of ethnic group and probability of marrying before 18 years while holding constant the socioeconomic condition of the family and the messages of early marriage prevention. As a proxy of family socioeconomic condition, we created a composite index of six household characteristics, where the higher the number of characteristics in the households, the higher the socioeconomic condition. When household characteristics are taken into account in Model 2, the significance level for the Mandinka ethnic group disappeared, while that for the Wolof ethnic group remains statistically significant. The lack of significance for the Mandinka ethnic group indicates that part of the effect observed in Model 1 was explained by the socioeconomic condition of the family and was not an effect of the Mandinka ethnic group by itself. On the other hand, the result of the Wolof ethnic group indicates that independent of the socioeconomic conditions, this ethnic group has a higher probability of marrying early.

In addition to the variables included in Model 2 and Model 3, we included the females’ duration of schooling. The inclusion of this variable will help to elucidate whether female education may confound the associations among ethnic group, family socioeconomic condition and marrying before 18 years. The results of Model 2 indicate that when family socioeconomic conditions and adolescent female education are held constant, females in the Wolof ethnic group have a 3-fold higher probability of marrying before 18 than do females in the Fula ethnic group.

In Model 4, we added five more variables to those evaluated in Model 3. The results show that when family socioeconomic condition, adolescent female education, living mother, living father, mother’s/father’s ability to read and write and whether the female adolescent agrees that fathers, as heads of household, and not mothers, should arrange for their daughters’ and sons’ marriage are held constant, females in the Wolof ethnic group still have nearly a 3-fold higher probability of marrying early than females in the Fula ethnic group. All these results indicate that ethnicity, particularly Wolof, exert an independent effect on the probability of marrying early. These results were crossed examined with findings from the focus group discussions and key informant interviews, which explored alternative explanations for early marriage.

***Themes identified during focus group discussions and key informant interviews.*** Other factors associated with early marriage also included the fear that girls will engage in premarital sex, in addition to lack of meaningful alternatives to marriage in rural areas.

**Figure 2.**
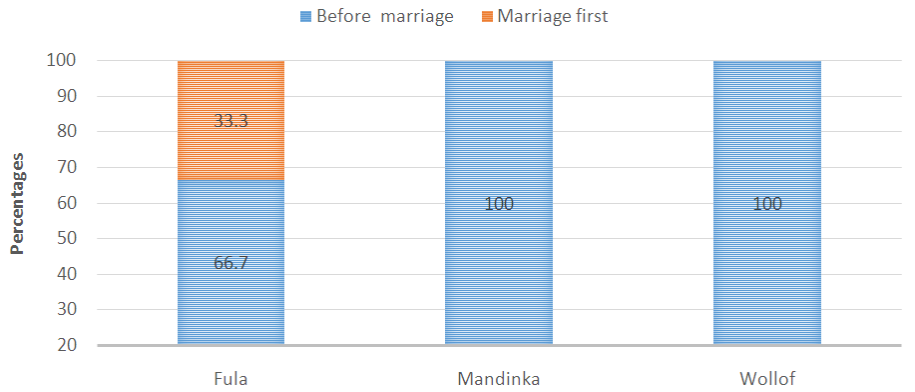
First sexual encounter and marriage: What came first?

***Fear of premarital sex.*** Findings from the focus group discussions revealed that the fear or concern that girls will engage in premarital sex, leading to loss of virginity before marriage or out-of-wedlock pregnancies is among the main reasons for practicing early marriage. In one focus group discussion, a participant, was quoted as saying: “ *It is very risky to delay marriage for your daughter because you don’t know what that might become of her*”. This statement was corroborated by another participant. The participant added the following: “ *In fact, in the first place, there is no guarantee that she [your daughter] is not having love affairs. To be sure that she does not have sexual encounter or loss her virginity before marriage, it is better to marry her off*”. From the above statements it is clear that fear of premarital sex is a major concern. This finding is supported by the survey data which indicate that among Mandinka and Wolof girls, the first sexual encounter generally occurred before marriage ( Figure 2). Therefore, considering that the first sexual encounter generally occurred before marriage among Mandinka and Wolof girls, it was accurate, as reported in the FGDs, that the fear that girls may engage in premarital sex is a major concern, leading parents to favor early marriage as a response strategy.

***Lack of meaningful alternatives to marriage.*** Participants’ narratives suggest that lack of meaningful alternatives to marriage including work opportunities also contribute to parents’ decision to marry off their daughters early. A village head was quoted as saying: *“My daughter, she could be used as an example. She graduated from grade twelve [High School] and later studied banking and finance. But still she could not find work. Imagine if she was able to find work, I would not have married her off early”.* A lady councilor also added: *“Taking care of a girl is not easy. If she has no work after she has finished school, you have to do everything for her including buying her clothes and giving her money to take care of her small needs.*

These and other related statements suggest that girls are often unable to find work even after they have completed secondary school and need to depend on their parents for survival. This may compel some parents to marry off their daughters early so as to reduce the burden on them. This situation may be more complicated in rural communities where there is perceived scarcity of “good” potential husbands, such as explained by this participant: *“There is great pressure on us [parents] to delay marriage for our girls. We at times submit to this pressure. But the thing is that a good husband is also hard to come by so that when this opportunity arises for your girl, you are left with no other options but to marry her off”*.

Taken together, these factors might at least partly explain the reasons for the continuing practice of early marriage in rural Gambian communities in Lower Baddibu District.

## Discussion

Our findings suggest that ethnicity and the fear that girls may engage in premarital sex are two important factors associated with early marriage in rural Gambia. We found that, ethnicity, particularly Wolof, exerts an independent effect on the probability of marrying early. The finding about Wolof ethnicity having an independent effect on the probability of marrying early is interesting because they are the only ethnic group that does not typically practice female genital cutting (FGC), and the fear or concern that girls may engage in premarital sex is among the main reasons for practicing both FGC and early marriage ^27^. This finding is also consistent with a study ^44^ that showed ethnic factors as determinants of early marriage among young females. Our finding about fear of premarital sex as a major factor in early wedlock is also in keeping with the chastity explanation as proposed by Bicchieri *et al.* ^45^, which explains that parents want their daughters to be chaste and believe that there is a risk that girls who grow older may lose their virginity outside of marriage. This finding has wide programmatic implications. It suggests that in order to decrease early marriages in rural Gambia, future programs should focus on allaying the fears around premarital sex. Such programs may include providing girls with age appropriate information to prevent early sexual activities and shifting social norms surrounding how and why family planning is used because currently family planning is practiced only for birth spacing rather than for delaying first pregnancy ^27^. Many participants also suggested providing girls with information about the effects of premarital sex and teenage pregnancies and organizing community engagement forums and discussion sessions with parents focusing on the social norms around early marriage.

We also found that lack of meaningful alternatives to marriage including work opportunities in rural areas may also limit the options and resources available to girl, resulting in early marriage. This finding suggest a need to provide girls with employment-oriented education including vocational skills which may result into more empowerment and a delay in marriage.

While our study has provided a contextualized picture of the underlying social and cultural factors that are perpetuating early marriage in rural Gambia, the findings should be interpreted in light of the following limitations. First, it was conducted in a single district, which limits generalization to other districts. There is evidence that study findings on early marriage from communities cannot be generalized to other countries or even to other communities within the country where the study was conducted ^25^. Second, because child marriage is illegal in the Gambia, participants may have provided measured responses for fear that they may be blamed by other community members and/or possibly be prosecuted by providing enough information on the situation of early marriage. Finally, although the study has touched on some important factors perpetuating early marriage, it does not provide enough qualitative data that could be used to explain differences in marriage patterns between ethnic groups. A deeper exploration of the specific factors that are relevant to the differences in marriage patterns between ethnic groups and the relationship between early marriage and female genital is merited. Nonetheless, the study finding has important implications for policy and or practice. It can be used by program managers to accelerate the decline of early marriages in rural Gambia.

## Data availability

### Underlying data

Figshare: Social and cultural factors perpetuating early marriage in rural Gambia: an exploratory mixed methods study. https://doi.org/10.6084/m9.figshare.10055516.v1 ^43^.

This project contains de-identified summary transcripts of focus group discussions. Focus group transcripts.

Figshare: Social and cultural factors perpetuating early marriage in rural Gambia: an exploratory mixed methods study. https://doi.org/10.6084/m9.figshare.11483385 ^42^

This project contains summary transcripts of key informant interviews

Figshare: Social and cultural factors perpetuating early marriage in rural Gambia: an exploratory mixed methods study. https://doi.org/10.6084/m9.figshare.10045979.v1 ^41^.

This project contains responses to the questionnaires administered in this study.

### Extended data

Figshare: Social and cultural factors perpetuating early marriage in rural Gambia: an exploratory mixed methods study. https://doi.org/10.6084/m9.figshare.10046024.v1 ^37^.

This project contains the focus group discussion guide.

Figshare: Social and cultural factors perpetuating early marriage in rural Gambia: an exploratory mixed methods study. https://doi.org/10.6084/m9.figshare.10046090.v1 ^34^.

This project contains the questionnaire administered to the female adolescents.

Figshare: Social and cultural factors perpetuating early marriage in rural Gambia: an exploratory mixed methods study. https://doi.org/10.6084/m9.figshare.10046102.v1 ^35^.

This project contains the household questionnaire.

Data are available under the terms of the Creative Commons Attribution 4.0 International license (CC-BY 4.0).
