# Supplementary material for: Social and cultural factors perpetuating early marriage in rural Gambia: an exploratory mixed methods study
Source: F1000Res. 2020 Jan 24;8:1949. Originally published 2019 Nov 21. [Version 3] doi: 10.12688/f1000research.21076.3 (PMC6974925; doi:10.12688/f1000research.21076.3)
Supplement: Peer review history

# Peer review: Reviewer response for version 2

**DOI:** 10.5256/f1000research.24239.r58513

**Published:** 2020-01-21

**Authors:** Peter J. Winch, Anna Passaniti

**Affiliations:** 1 Department of International Health, Johns Hopkins Bloomberg School of Public Health, Baltimore, MD, USA; 2 School of Public Health, Johns Hopkins Bloomberg School of Public Health, Baltimore, MD, USA

Article is approved

 We still think this is an important point to integrate somewhere in the Discussion:

- The finding about Wolof ethnicity having an independent effect on the probability of marrying early is interesting because they are the only ethnic group that does not typically practice female genital cutting (FGC), and the fear/concern that girls will engage in premarital sex is among the main reasons for practicing both FGC and early marriage.

We confirm that we have read this submission and believe that we have an appropriate level of expertise to confirm that it is of an acceptable scientific standard.

# Peer review: Reviewer response for version 1

**DOI:** 10.5256/f1000research.23195.r57612

**Published:** 2019-12-24

**Authors:** Peter J. Winch, Anna Passaniti

**Affiliations:** 1 Department of International Health, Johns Hopkins Bloomberg School of Public Health, Baltimore, MD, USA; 2 School of Public Health, Johns Hopkins Bloomberg School of Public Health, Baltimore, MD, USA

**Abstract:**

- Conclusions repetitive of the Results section of the Abstract.
- Results and Conclusions sections not well integrated, and are not very specific. What about ethnicity factors early marriage? What are the social and cultural norms? Much repetition between the two sections of the Abstract. Does not create excitement in the reader to download and read this paper.

- Results: Using multiple regression analysis, this study found that ethnicity more than other factors, exerts an independent effect on early marriage. Themes identified during focus group discussions also revealed that fear of premarital sex and loss of virginity outside marriage were major reasons for the perpetuation of early marriage.
- Conclusions: These findings suggest that the practice of early marriage in rural Gambia is associated with ethnicity and practices related to social and cultural norms. The findings also suggest that in order to decrease early marriages, future efforts should focus on allaying the fears around premarital sex and loss of virginity related to delay in marriage.

**Introduction:**

- Good that they presented a universal definition of child marriage at the very beginning.
- What are the consequences of early marriage that the authors are particularly concerned with? Is it from a public health lens, access to education, human rights, bodily autonomy, gender equality? Explicitly stating *why and how *child marriage is a problem in West Africa would strengthen this section.

**Early Marriage in The Gambia: **

- It might be necessary to note that many women and adolescents of childbearing age from rural Gambia are only recently getting birth certificates, where their age is estimated from memory since the date wasn’t recorded on their actual date of birth.
- Especially for women who need a birth certificate to access an antenatal card, We have observed that since now most everyone knows that child marriage is illegal, they report being 18 years of age when in actuality they are much younger. Therefore, the reported rate of 30% here is unlikely to be accurate.
- High awareness but low uptake of family planning among adolescents: A major factor seems to be people’s preference to use family planning only for birth spacing, rather than for delaying first pregnancy.

**Methods:**

- In the first paragraph on page 6, rephrase: “Parental informed consent was sought for minors under the age of 18 years. Since the research team was composed of men, permission was also taken from husbands of married female adolescents because culturally, husbands are expected  to give permission for other men to speak with their wives.”
- The qualitative component of the study is minimal. With only two focus groups, the authors are not in a position to explain differences in marriage patterns between ethnic groups. If this were a major objective of the study, in-depth interviews with different family members including elders would be necessary. Therefore, it is unclear what is gained from the focus groups. Certainly, only limited qualitative data are presented in the Results section.
- The description of the qualitative data analysis is very generic.

**Results:**

- Good break down of demographic characteristics. The rationale behind why these characteristics were selected is also strong and clear.
- The finding about Wolof ethnicity having an independent effect on the probability of marrying early is interesting because they are the only ethnic group that does not typically practice female genital cutting (FGC), and the fear/ concern that girls will engage in premarital sex is among the main reasons for practicing both FGC and early marriage.
- This study brings up further questions about the relationship between practicing early marriage and practicing FGC – for example, are groups that do not practice FGC more likely to practice early marriage because they are not using FGC as a tool to prevent premarital sex? This points again to the fact that conducting only two focus groups, and no interviews, provides little insight into the situation. The authors do not present qualitative data that explain the reasons for differences between the ethnic groups with any detail or clarity.

**Discussion:**

- In the “Early Marriage in The Gambia” section, the writers brought up that awareness of family planning methods is high but uptake is low among adolescents. The findings may suggest that shifting socials norms surrounding how and why family planning is used (i.e. currently  only for birth spacing rather than delaying first pregnancy) may also be an important programmatic implication.
- The weakness and limited nature of the qualitative data collection make it difficult to arrive at robust conclusions.

We confirm that we have read this submission and believe that we have an appropriate level of expertise to confirm that it is of an acceptable scientific standard, however we have significant reservations, as outlined above.

# Peer review: Reviewer response for version 1

**DOI:** 10.5256/f1000research.23195.r56901

**Published:** 2019-12-17

**Authors:** Oluwaseyi Abiodun Akpor

**Affiliations:** 1 Department of Nursing Science, College of Medicine and Health Sciences, Afe Babalola University, Ado Ekiti, Nigeria

The study was aimed at providing information on the type and short-term effects of contextually relevant interventions that can be scaled up to change societal attitudes towards accelerating the decline of early marriages in rural Gambia. Overall, the study design was appropriate with robust data collection and interpretation. The data were adequately interpreted and discussed.

 The authors should however reduce the length of the background in the abstract and include more findings in the result section.

 The manuscript is recommended for indexing, subject to the minor revision.

I confirm that I have read this submission and believe that I have an appropriate level of expertise to confirm that it is of an acceptable scientific standard.
